# Supplementary material for: A Critical View of the Application of the APEX Software (Aqueous Photochemistry of Environmentally-Occurring Xenobiotics) to Predict Photoreaction Kinetics in Surface Freshwaters
Source: Molecules. 2019 Dec 18;25(1):9. doi: 10.3390/molecules25010009 (PMC7017383; doi:10.3390/molecules25010009)
Supplement: Supplementary file 1 [file molecules-25-00009-s001.zip › 3.2.4_gcc-4.4.0.zip!/3.2.4_gcc-4.4.0/3.2.4_gcc-4.4.0/doc/octave/html/interpreter/Distributions.html]

Distributions - Untitled


Next: Random Number Generation,
Previous: Models,
Up: Statistics


---

### 25.6 Distributions

Octave has functions for computing the Probability Density Function
(PDF), the Cumulative Distribution function (CDF), and the quantile
(the inverse of the CDF) of a large number of distributions.

The following table summarizes the supported distributions (in
alphabetical order).

|  |  |  |  |
| --- | --- | --- | --- |
| **Distribution** | **PDF** | **CDF** | **Quantile** |
| Beta Distribution | `betapdf` | `betacdf` | `betainv` |
| Binomial Distribution | `binopdf` | `binocdf` | `binoinv` |
| Cauchy Distribution | `cauchy_pdf` | `cauchy_cdf` | `cauchy_inv` |
| Chi-Square Distribution | `chi2pdf` | `chi2cdf` | `chi2inv` |
| Univariate Discrete Distribution | `discrete_pdf` | `discrete_cdf` | `discrete_inv` |
| Empirical Distribution | `empirical_pdf` | `empirical_cdf` | `empirical_inv` |
| Exponential Distribution | `exppdf` | `expcdf` | `expinv` |
| F Distribution | `fpdf` | `fcdf` | `finv` |
| Gamma Distribution | `gampdf` | `gamcdf` | `gaminv` |
| Geometric Distribution | `geopdf` | `geocdf` | `geoinv` |
| Hypergeometric Distribution | `hygepdf` | `hygecdf` | `hygeinv` |
| Kolmogorov Smirnov Distribution | *Not Available* | `kolmogorov_smirnov_cdf` | *Not Available* |
| Laplace Distribution | `laplace_pdf` | `laplace_cdf` | `laplace_inv` |
| Logistic Distribution | `logistic_pdf` | `logistic_cdf` | `logistic_inv` |
| Log-Normal Distribution | `lognpdf` | `logncdf` | `logninv` |
| Pascal Distribution | `nbinpdf` | `nbincdf` | `nbininv` |
| Univariate Normal Distribution | `normpdf` | `normcdf` | `norminv` |
| Poisson Distribution | `poisspdf` | `poisscdf` | `poissinv` |
| t (Student) Distribution | `tpdf` | `tcdf` | `tinv` |
| Univariate Discrete Distribution | `unidpdf` | `unidcdf` | `unidinv` |
| Uniform Distribution | `unifpdf` | `unifcdf` | `unifinv` |
| Weibull Distribution | `wblpdf` | `wblcdf` | `wblinv` |

— Function File: **betacdf** (x, a, b)  
> For each element of x, returns the CDF at x of the beta
> distribution with parameters a and b, i.e.,
> PROB (beta (a, b) <= x).

— Function File: **betainv** (x, a, b)  
> For each component of x, compute the quantile (the inverse of
> the CDF) at x of the Beta distribution with parameters a
> and b.

— Function File: **betapdf** (x, a, b)  
> For each element of x, returns the PDF at x of the beta
> distribution with parameters a and b.

— Function File: **binocdf** (x, n, p)  
> For each element of x, compute the CDF at x of the
> binomial distribution with parameters n and p.

— Function File: **binoinv** (x, n, p)  
> For each element of x, compute the quantile at x of the
> binomial distribution with parameters n and p.

— Function File: **binopdf** (x, n, p)  
> For each element of x, compute the probability density function
> (PDF) at x of the binomial distribution with parameters n
> and p.

— Function File: **cauchy\_cdf** (x, lambda, sigma)  
> For each element of x, compute the cumulative distribution
> function (CDF) at x of the Cauchy distribution with location
> parameter lambda and scale parameter sigma. Default
> values are lambda = 0, sigma = 1.

— Function File: **cauchy\_inv** (x, lambda, sigma)  
> For each element of x, compute the quantile (the inverse of the
> CDF) at x of the Cauchy distribution with location parameter
> lambda and scale parameter sigma. Default values are
> lambda = 0, sigma = 1.

— Function File: **cauchy\_pdf** (x, lambda, sigma)  
> For each element of x, compute the probability density function
> (PDF) at x of the Cauchy distribution with location parameter
> lambda and scale parameter sigma > 0. Default values are
> lambda = 0, sigma = 1.

— Function File: **chi2cdf** (x, n)  
> For each element of x, compute the cumulative distribution
> function (CDF) at x of the chisquare distribution with n
> degrees of freedom.

— Function File: **chi2inv** (x, n)  
> For each element of x, compute the quantile (the inverse of the
> CDF) at x of the chisquare distribution with n degrees of
> freedom.

— Function File: **chisquare\_pdf** (x, n)  
> For each element of x, compute the probability density function
> (PDF) at x of the chisquare distribution with n degrees
> of freedom.

— Function File: **discrete\_cdf** (x, v, p)  
> For each element of x, compute the cumulative distribution
> function (CDF) at x of a univariate discrete distribution which
> assumes the values in v with probabilities p.

— Function File: **discrete\_inv** (x, v, p)  
> For each component of x, compute the quantile (the inverse of
> the CDF) at x of the univariate distribution which assumes the
> values in v with probabilities p.

— Function File: **discrete\_pdf** (x, v, p)  
> For each element of x, compute the probability density function
> (PDF) at x of a univariate discrete distribution which assumes
> the values in v with probabilities p.

— Function File: **empirical\_cdf** (x, data)  
> For each element of x, compute the cumulative distribution
> function (CDF) at x of the empirical distribution obtained from
> the univariate sample data.

— Function File: **empirical\_inv** (x, data)  
> For each element of x, compute the quantile (the inverse of the
> CDF) at x of the empirical distribution obtained from the
> univariate sample data.

— Function File: **empirical\_pdf** (x, data)  
> For each element of x, compute the probability density function
> (PDF) at x of the empirical distribution obtained from the
> univariate sample data.

— Function File: **expcdf** (x, lambda)  
> For each element of x, compute the cumulative distribution
> function (CDF) at x of the exponential distribution with
> mean lambda.
>
> The arguments can be of common size or scalar.

— Function File: **expinv** (x, lambda)  
> For each element of x, compute the quantile (the inverse of the
> CDF) at x of the exponential distribution with mean
> lambda.

— Function File: **exppdf** (x, lambda)  
> For each element of x, compute the probability density function
> (PDF) of the exponential distribution with mean lambda.

— Function File: **fcdf** (x, m, n)  
> For each element of x, compute the CDF at x of the F
> distribution with m and n degrees of freedom, i.e.,
> PROB (F (m, n) <= x).

— Function File: **finv** (x, m, n)  
> For each component of x, compute the quantile (the inverse of
> the CDF) at x of the F distribution with parameters m and
> n.

— Function File: **fpdf** (x, m, n)  
> For each element of x, compute the probability density function
> (PDF) at x of the F distribution with m and n
> degrees of freedom.

— Function File: **gamcdf** (x, a, b)  
> For each element of x, compute the cumulative distribution
> function (CDF) at x of the Gamma distribution with parameters
> a and b.
>
>
>
> **See also:** gamma, gammaln, gammainc, gampdf, gaminv, gamrnd.

— Function File: **gaminv** (x, a, b)  
> For each component of x, compute the quantile (the inverse of
> the CDF) at x of the Gamma distribution with parameters a
> and b.
>
>
>
> **See also:** gamma, gammaln, gammainc, gampdf, gamcdf, gamrnd.

— Function File: **gampdf** (x, a, b)  
> For each element of x, return the probability density function
> (PDF) at x of the Gamma distribution with parameters a
> and b.
>
>
>
> **See also:** gamma, gammaln, gammainc, gamcdf, gaminv, gamrnd.

— Function File: **geocdf** (x, p)  
> For each element of x, compute the CDF at x of the
> geometric distribution with parameter p.

— Function File: **geoinv** (x, p)  
> For each element of x, compute the quantile at x of the
> geometric distribution with parameter p.

— Function File: **geopdf** (x, p)  
> For each element of x, compute the probability density function
> (PDF) at x of the geometric distribution with parameter p.

— Function File: **hygecdf** (x, t, m, n)  
> Compute the cumulative distribution function (CDF) at x of the
> hypergeometric distribution with parameters t, m, and
> n. This is the probability of obtaining not more than x
> marked items when randomly drawing a sample of size n without
> replacement from a population of total size t containing
> m marked items.
>
> The parameters t, m, and n must positive integers
> with m and n not greater than t.

— Function File: **hygeinv** (x, t, m, n)  
> For each element of x, compute the quantile at x of the
> hypergeometric distribution with parameters t, m, and
> n.
>
> The parameters t, m, and n must positive integers
> with m and n not greater than t.

— Function File: **hygepdf** (x, t, m, n)  
> Compute the probability density function (PDF) at x of the
> hypergeometric distribution with parameters t, m, and
> n. This is the probability of obtaining x marked items
> when randomly drawing a sample of size n without replacement
> from a population of total size t containing m marked items.
>
> The arguments must be of common size or scalar.

— Function File: **kolmogorov\_smirnov\_cdf** (x, tol)  
> Return the CDF at x of the Kolmogorov-Smirnov distribution,
>
> ```
>                    Inf
>           Q(x) =   SUM    (-1)^k exp(-2 k^2 x^2)
>                  k = -Inf
> ```
>
> for x > 0.
>
> The optional parameter tol specifies the precision up to which
> the series should be evaluated; the default is tol = `eps`.

— Function File: **laplace\_cdf** (x)  
> For each element of x, compute the cumulative distribution
> function (CDF) at x of the Laplace distribution.

— Function File: **laplace\_inv** (x)  
> For each element of x, compute the quantile (the inverse of the
> CDF) at x of the Laplace distribution.

— Function File: **laplace\_pdf** (x)  
> For each element of x, compute the probability density function
> (PDF) at x of the Laplace distribution.

— Function File: **logistic\_cdf** (x)  
> For each component of x, compute the CDF at x of the
> logistic distribution.

— Function File: **logistic\_inv** (x)  
> For each component of x, compute the quantile (the inverse of
> the CDF) at x of the logistic distribution.

— Function File: **logistic\_pdf** (x)  
> For each component of x, compute the PDF at x of the
> logistic distribution.

— Function File: **logncdf** (x, mu, sigma)  
> For each element of x, compute the cumulative distribution
> function (CDF) at x of the lognormal distribution with
> parameters mu and sigma. If a random variable follows this
> distribution, its logarithm is normally distributed with mean
> mu and standard deviation sigma.
>
> Default values are mu = 1, sigma = 1.

— Function File: **logninv** (x, mu, sigma)  
> For each element of x, compute the quantile (the inverse of the
> CDF) at x of the lognormal distribution with parameters mu
> and sigma. If a random variable follows this distribution, its
> logarithm is normally distributed with mean `log (`mu`)` and
> variance sigma.
>
> Default values are mu = 1, sigma = 1.

— Function File: **lognpdf** (x, mu, sigma)  
> For each element of x, compute the probability density function
> (PDF) at x of the lognormal distribution with parameters
> mu and sigma. If a random variable follows this distribution,
> its logarithm is normally distributed with mean mu
> and standard deviation sigma.
>
> Default values are mu = 1, sigma = 1.

— Function File: **nbincdf** (x, n, p)  
> For each element of x, compute the CDF at x of the Pascal
> (negative binomial) distribution with parameters n and p.
>
> The number of failures in a Bernoulli experiment with success
> probability p before the n-th success follows this
> distribution.

— Function File: **nbininv** (x, n, p)  
> For each element of x, compute the quantile at x of the
> Pascal (negative binomial) distribution with parameters n and
> p.
>
> The number of failures in a Bernoulli experiment with success
> probability p before the n-th success follows this
> distribution.

— Function File: **nbinpdf** (x, n, p)  
> For each element of x, compute the probability density function
> (PDF) at x of the Pascal (negative binomial) distribution with
> parameters n and p.
>
> The number of failures in a Bernoulli experiment with success
> probability p before the n-th success follows this
> distribution.

— Function File: **normcdf** (x, m, s)  
> For each element of x, compute the cumulative distribution
> function (CDF) at x of the normal distribution with mean
> m and standard deviation s.
>
> Default values are m = 0, s = 1.

— Function File: **norminv** (x, m, s)  
> For each element of x, compute the quantile (the inverse of the
> CDF) at x of the normal distribution with mean m and
> standard deviation s.
>
> Default values are m = 0, s = 1.

— Function File: **normpdf** (x, m, s)  
> For each element of x, compute the probability density function
> (PDF) at x of the normal distribution with mean m and
> standard deviation s.
>
> Default values are m = 0, s = 1.

— Function File: **poisscdf** (x, lambda)  
> For each element of x, compute the cumulative distribution
> function (CDF) at x of the Poisson distribution with parameter
> lambda.

— Function File: **poissinv** (x, lambda)  
> For each component of x, compute the quantile (the inverse of
> the CDF) at x of the Poisson distribution with parameter
> lambda.

— Function File: **poisspdf** (x, lambda)  
> For each element of x, compute the probability density function
> (PDF) at x of the poisson distribution with parameter lambda.

— Function File: **tcdf** (x, n)  
> For each element of x, compute the cumulative distribution
> function (CDF) at x of the t (Student) distribution with
> n degrees of freedom, i.e., PROB (t(n) <= x).

— Function File: **tinv** (x, n)  
> For each probability value x, compute the inverse of the
> cumulative distribution function (CDF) of the t (Student)
> distribution with degrees of freedom n. This function is
> analogous to looking in a table for the t-value of a single-tailed
> distribution.

— Function File: **tpdf** (x, n)  
> For each element of x, compute the probability density function
> (PDF) at x of the t (Student) distribution with n
> degrees of freedom.

— Function File: **unidcdf** (x, v)  
> For each element of x, compute the cumulative distribution
> function (CDF) at x of a univariate discrete distribution which
> assumes the values in v with equal probability.

— Function File: **unidinv** (x, v)  
> For each component of x, compute the quantile (the inverse of
> the CDF) at x of the univariate discrete distribution which assumes the
> values in v with equal probability

— Function File: **unidpdf** (x, v)  
> For each element of x, compute the probability density function
> (PDF) at x of a univariate discrete distribution which assumes
> the values in v with equal probability.

— Function File: **unifcdf** (x, a, b)  
> Return the CDF at x of the uniform distribution on [a,
> b], i.e., PROB (uniform (a, b) <= x).
>
> Default values are a = 0, b = 1.

— Function File: **unifinv** (x, a, b)  
> For each element of x, compute the quantile (the inverse of the
> CDF) at x of the uniform distribution on [a, b].
>
> Default values are a = 0, b = 1.

— Function File: **unifpdf** (x, a, b)  
> For each element of x, compute the PDF at x of the uniform
> distribution on [a, b].
>
> Default values are a = 0, b = 1.

— Function File: **wblcdf** (x, scale, shape)  
> Compute the cumulative distribution function (CDF) at x of the
> Weibull distribution with shape parameter scale and scale
> parameter shape, which is
>
> ```
>           1 - exp(-(x/shape)^scale)
> ```
>
> for x >= 0.

— Function File: **wblinv** (x, scale, shape)  
> Compute the quantile (the inverse of the CDF) at x of the
> Weibull distribution with shape parameter scale and scale
> parameter shape.

— Function File: **wblpdf** (x, scale, shape)  
> Compute the probability density function (PDF) at x of the
> Weibull distribution with shape parameter scale and scale
> parameter shape which is given by
>
> ```
>              scale * shape^(-scale) * x^(scale-1) * exp(-(x/shape)^scale)
> ```
>
> for x > 0.
